# Arrowroot Starch/ZnO Nanoparticle Nanocomposite Films: Thermal, Morphological, and Tensile Properties

**DOI:** 10.1021/acsomega.5c00255

**Published:** 2025-06-30

**Authors:** Bianca Correa Pinto, Natan Gabriel da Silva Nunes, Alcy Favacho Ribeiro, Bruno Marques Viegas, José Antonio da Silva Souza, Jordan Del Nero, Waldomiro Gomes Paschoal Júnior, Severino Alves Júnior, Emanuel Negrão Mâcedo, Verônica Scarpini Cândido, Carlos Alberto Brito da Silva, Marcos Vinícius da Silva Paula, Ana Aurea Barreto Maia

**Affiliations:** a Programa de Pós-Graduação em Ciência e Engenharia de Materiais, Universidade Federal do Pará, Ananindeua 67130-660, Brazil; b Faculdade de Engenharia de Materiais,Universidade Federal do Pará, Ananindeua 67130-660, Brazil; c Faculdade de Química, Universidade Federal do Pará, Ananindeua 67130-660, Brazil; d Programa de Pós-Graduação em Biotecnologia, Universidade Federal do Pará, Belém 66075-110, Brazil; e Programa de Pós-Graduação em Engenharia de Recursos Naturais da Amazônia, Universidade Federal do Pará, Belém 66079-420, Brazil; f Faculdade de Física, Universidade Federal do Pará, Belém 66075-110, Brazil; g Departamento de Química Fundamental, Universidade Federal de Pernambuco, Recife 50740-560, Brazil; h Faculdade de Física, Universidade Federal do Pará, Ananindeua 67130-660, Brazil

## Abstract

Petroleum-derived conventional plastics play an important role in our lives despite their accumulation in the environment. An alternative is natural polymers, in which starch has been highlighted. However, starch-derived films exhibit a low tensile strength. One way to improve this is by adding zinc oxide nanoparticles (ZnO NPs). In this work, thermoplastic arrowroot starch/ZnO nanocomposite (TPA/ZnO NC) films were manufactured by solvent casting, and their thermal, tensile, morphological, and soil burial degradation properties were investigated. In addition, the TPA/ZnO NC films were evaluated for their suitability for bread packaging. TEM confirmed the nanometer dimension for ZnO. XRD confirmed the formation of a thermoplastic material with characteristic peaks of ZnO NPs, but characteristic bands for ZnO were not observed in the FTIR. SEM images showed that ZnO NP concentrations are randomly distributed in the TPA matrix. The ZnO NPs did not cause major changes in the thermal degradation profile of the NC films in relation to the starch matrix, as demonstrated by TGA results. TPA and TPA/ZnO-5 films exhibited an improvement in tensile strength (0.62–1.44 MPa) and modulus (1.73–10.46 MPa), respectively. ZnO NPs modified the moisture, solubility, and transparency of the films. Our results demonstrate that soil burial degradation for the films depends on the soil moisture. Black and yellow spots, characteristic of fungal growth, were not observed on bread stored in the TPA/ZnO-3 and TPA/ZnO-5 films. Our results indicate that TPA/ZnO-5 demonstrated a favorable combination of σ, ε, and *E* and also exhibited soil burial degradation.

## Graphical abstract

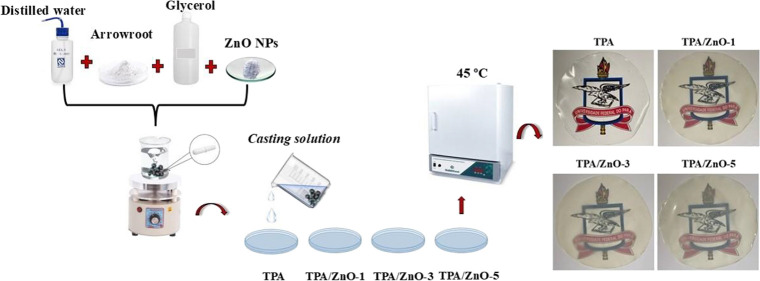

## 1 Introduction

Conventional plastics derived from petroleum play an essential role in our lives due to their desirable properties, including light weight, weather resistance, low cost, ease of transportation, and transparency. However, one of the main disadvantages of these materials is their long degradation time, which has led to their accumulation in the environment. In this context, considering the environmental impact and the consequences of the long decomposition time of petroleum plastics, the search for biodegradable materials is essential.^1–3^

Biodegradable materials are decomposed by natural biological agents, avoiding water and soil pollution.^4,5^ Among biodegradable materials, natural polymers such as starch are a solution to the long degradation time of petroleum-derived plastics.^6,7^ Starch is the most used natural polymer for developing biodegradable products due to its characteristics such as abundance, low cost, and biodegradation.^8,9^ Starch is made up of granules that contain a mixture of two compounds: amylose and amylopectin, which can vary according to the characteristics and proportions defined by each plant origin. In other words, granular starch is formed by two homopolysaccharides (amylose and amylopectin).^10–12^

Thermoplastic starch (TPS) is a product resulting from the combination of starch with the addition of a plasticizer (i.e., water and/or glycerol) with subsequent processing by heating and continuous shearing.^13^ This processing results in a homogeneous, continuous, and viscous phase.^14^ The amylose and amylopectin chains are intercalated in this molten phase, while the semicrystalline structure of the starch granule is destroyed.^15^ TPS can be processed by traditional thermoplastic processing techniques: gelatinization in an aqueous medium, injection, or extrusion.^16^ The final properties of starch depend on the choice of additives and the processing conditions. In general, TPS has low mechanical strength and high permeability to gases and vapors, which restrict its use in the food packaging sector.^17^

Thus, arrowroot (*Maranta arundinacea*), a species native to South America, appears as an alternative source of starch with approximately 35% amylose content, which contributes to the formation of films with better traction and barrier properties. Arrowroot is a perennial plant with a height ranging from 90 to 150 cm. Its characteristics include white flowers, green leaves with a length of 10 to 20 cm, and thick rhizomes with a width of 2.5 to 3 cm and a length of 20 to 40 cm. Arrowroot rhizomes are typically found in clusters of two or three, although they can also appear isolated.^15,18,19^ Arrowroot is a vegetable with a significant starch content, carefully extracted from its rhizomes through a process of grinding and decantation.^20^ In this way, the use of thermoplastic arrowroot starch is highly promising for use in the food packaging sector. However, the use of thermoplastic arrowroot starch in the food packaging sector still presents traditional disadvantages of starch films, such as low tensile strength.

Active packaging is a type of material that, after the addition of additives, maintains the quality and extends the shelf life of food without losing its integrity.^21,22^ For applications in the food packaging sector, biopolymers are quite attractive, as these materials are nontoxic, odorless, and edible.^23^ Antimicrobial agents are one of the most studied chemical agents for use in food packaging, as they act by inhibiting the growth of microorganisms that are responsible for food degradation.^24^ Among the antimicrobial agents, zinc oxide (ZnO) nanoparticles (NPs) stand out. ZnO is a semiconductor that attracts attention due to its wide applicability in the biomedical area, with emphasis on its antimicrobial and antifungal activity.^25,26^ Its antimicrobial activity is significantly enhanced by its smaller size and greater surface area.^27^ The addition of ZnO NPs to the arrowroot starch matrix aims to provide antimicrobial activity to the final product.^7^ This addition (NP) to a polymer matrix results in a material called a polymer nanocomposite (NC).^28^ Furthermore, the addition of these NPs to the arrowroot starch matrix can act to improve the tensile strength of starch films. Recently, several studies have been reported in the literature on the use of ZnO NPs in polymeric matrices for applications in the food packaging sector. In a previous study, ZnO NPs modified with poly­(vinyl alcohol) were added into polycaprolactone films. The authors reported that the tensile strength of the films was improved after the addition of NPs.^29^ Zhu et al. fabricated starch nanocomposite (NC) films via extrusion blowing, incorporating nano-ZnO and nano-SiO_2_. The films exhibited improved thermal, mechanical, and moisture barrier properties due to the addition of these nanomaterials.^30^

In the same way, Kodsangma et al. found for thermoplastic-modified starch (TPMS)/carboxymethyl cellulose (CMC) NC films with ZnO NPs prepared by reactive blending an improvement in mechanical properties and water resistance.^31^ Recently, Li et al. developed CMC/starch films with ZnO NPs and anthocyanins, and the films obtained exhibited improved ductility, water resistance, light barrier, and antimicrobial activity against *Escherichia coli* and *Staphylococcus aureus*.^7^ NCs of polybutylene adipate-*co*-terephthalate (PBAT) and starch blended ZnO NPs manufactured by blown extrusion enhanced the shelf life of meat by more than 72 h.^32^

To the best of our knowledge, there have been no investigations on the fabrication and characterization of arrowroot starch films with ZnO nanoparticles. Thus, in this study, arrowroot starch NC films with 0, 1, 3, and 5% of ZnO NPs were obtained by solvent casting, which is a standard, simple, and easy method, using glycerol as a plasticizer for applications in the food packaging sector as green food packaging films. The arrowroot starch/ZnO NP NC films were evaluated for their thermal, tensile, morphological, moisture, solubility, swelling, and transparency properties and soil burial degradation, showing significant improvements compared to those of the arrowroot starch film.

## 2 Experiments

### 2.1 Materials

The materials used in this study were arrowroot starch (*Maranta arundinacea*) supplied by Torres Company, São Paulo (SP), Brazil; glycerol PA ACS 97% supplied by Exodo Cientficain Sumaré-SP; distilled water kindly provided from the catalysis and biocatalysis laboratory (LABCAT- UFPA); and ZnO NPs supplied by Sigma-Aldrich. All reagents were used without prior treatment, and the description of the material used in this study is listed in Table 1, as well as the composition of reagents and their origin.

**Table 1:** Materials, Composition, and Supplier Used in the Preparation of the TPA and TPA/ZnO NC Films

materials	composition	supplier
arrowroot starch		Torres Company
glycerol P.A. ACS 97%	C_3_H_8_O_3_	Êxodo Científica
ZnO NPs < 100 nm	ZnO	Sigma-Aldrich

### 2.2 Methodology

#### 2.2.1 NC Film preparation

TPA starch films with 1, 3, and 5% of ZnO NPs (TPA, TPA1%, TPA3%, and TPA5% or TPA, TPA/ZnO-1, TPA/ZnO-3, and TPA/ZnO-5) were obtained by the solvent casting method as described by Fakhouri et al. with prior modifications. Previously established amounts of ZnO NPs were added to 20 mL of distilled water and stirred for 30 min. Then, arrowroot starch and glycerol were homogenized in distilled water added to the ZnO dispersion and kept at 80 °C for 10 min. The film-forming solutions were poured into Petri dishes with size 140 × 15 mm and kept at 45 °C for 24 h. The films were stored in polyethylene bags and placed in a desiccator with silica to avoid humidity. Figure 1 presents the methodology used for film preparation. The samples, reagents, and composition for the films are presented in Table 2.

**Figure 1:**
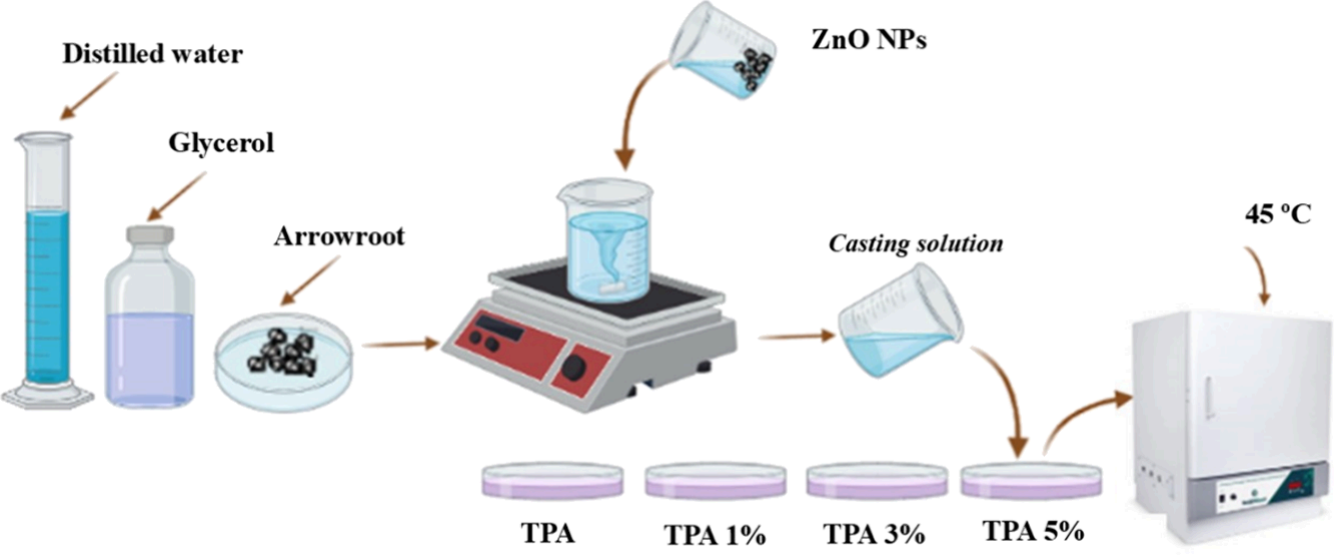
Scheme shows the methodology used to prepare NC films.

**Table 2:** Samples, Reagents, and Composition of NC Films

samples	reagents	composition
TPA	arrowroot starch, glycerol, H_2_O	4 g of arrowroot starch, 1.2 g of glycerol, and 80 mL of H_2_O
TPA/ZnO-1 (TPA 1%)	arrowroot starch, glycerol, H_2_O, and ZnO NPs < 100 nm	4 g of arrowroot starch, 1.2 g of glycerol, 80 mL of H_2_O, and 0.04 g of ZnO NPs
TPA/ZnO-3 (TPA 3%)	arrowroot starch, glycerol, H_2_O, and ZnO NPs < 100 nm	4 g of arrowroot starch, 1.2 g of glycerol, 80 mL of H_2_O, and 0.12 g of ZnO NPs
TPA/ZnO-5 (TPA 5%)	arrowroot starch, glycerol, H_2_O, and ZnO NPs < 100 nm	4 g of arrowroot starch, 1.2 g of glycerol, 80 mL of H_2_O, and 0.2 g of ZnO NPs

#### 2.2.2 Fourier Transform Infrared Spectroscopy (FTIR)

To evaluate the functional groups of the films, absorption spectra were obtained in the infrared region on a BRUKER model VERTEX 70v. FTIR spectra for the films were obtained by attenuated total reflectance (ATR) in the range of 4000–400 cm^–1^ at 100 scans and a resolution of 8 cm^–1^.

#### 2.2.3 Thermogravimetric Analysis (TGA)

TGA curves for the films were obtained on a simultaneous thermogravimetric analyzer from STA (model NEXTA STA 300). The curves were determined in an inert nitrogen atmosphere with a flow rate of 100 mL min^–1^ from room temperature to 800 °C with a heating rate of 10 °C min^–1^.

#### 2.2.4 X-ray Diffraction (XRD)

Diffractograms for TPA and NC films were acquired using a Rigaku Smart Lab X-ray diffractometer with Cu Kα radiation (1.54056 Å) at 40 kV and 30 mA. Diffractograms were collected between 5 and 60°, with a step of 0.02° and a fixed acquisition time of 1 s.

#### 2.2.5 Transmission Electron Microscopy (TEM)

The morphological characterization of ZnO NPs was carried out using a transmission electron microscope (Jeol, model JEM-2100) at 200 kV. ZnO NPs were suspended in water and then deposited on copper grids.

#### 2.2.6 Scanning Electron Microscopy (SEM)

SEM images were acquired to investigate the surface of starch grains, ZnO NPS, and TPA/ZnO NCs. First, the samples were adhered with carbon tapes to aluminum supports (stubs) and then metalized with a 10–20 nm thick gold layer using an Emitech metallizer model K675X Sputter Coater. SEM images were acquired using a TESCAN Mira3 scanning electron microscope operating at a voltage of 5 kV.

#### 2.2.7 Film Thickness

The average thickness was calculated from the measurement of five random points on each sample using a digital micrometer from SYNTEK (model 24601) with a measuring capacity of 0 cm to 25 mm and a resolution of 0.001 mm.

#### 2.2.8 Moisture Content

The moisture content was determined by the gravimetric method. The samples were cut into 2 × 2 cm pieces and then weighed. The final mass was recorded after drying the films at 105 °C for 24 h. The tests were conducted in triplicate for each type of film, and the moisture content [MC (%)] was calculated by eq 1:

$$\mathrm{MC}\;(\%)=\left(\frac{m_{i}-m_{f}}{m_{i}}\right)\times 100$$ 1

where *m*_i_ is the initial mass and *m*_f_ is the final mass.

#### 2.2.9 Solubility

Films with dimensions of 1.5 × 4 cm were dried at 100 °C for 24 h and then weighed. After this step, the dehydrated samples were immersed in 50 mL of distilled water for 24 h at room temperature. After this period, the excess water was removed, and the samples were again dried at 105 °C for 24 h and weighed again. The tests were conducted in triplicate for each type of sample, and the solubility in water [S (%)] was calculated according to eq 2:^34^

$$S\;(\%)=\left(\frac{m_{i}-m_{f}}{m_{i}}\right)\times 100$$ 2

where *m*_i_ is the initial mass and *m*_f_ is the final mass.

#### 2.2.10 Transparency

The transparency (Tr) of the samples was analyzed by a UV–vis spectrophotometer by acquiring the percentage of transmittance (*T%*) at 600 nm and determined by eq 3:^34^

$$\mathrm{Tr}=\frac{\log(T\%)}{y}$$ 3

where *y* is the thickness of the sample.

#### 2.2.11 Swelling in Water

To determine the swelling in water, films with dimensions of 2 × 2 cm were initially weighed. Subsequently, the samples were immersed in 100 mL of distilled water,at room temperature for previously predetermined times (60, 120, and 180 min). After the end of the immersion time, the samples were removed, dried with paper, and weighed again. The tests were performed in triplicate for each type of film, and the swelling in water [SW (%)] was given by eq 4:

$$\mathrm{SW}\;(\%)=\left(\frac{m_{f}-m_{i}}{m_{i}}\right)\times 100$$ 4

where *m*_i_ is the initial mass and *m*_f_ is the final mass.

#### 2.2.12 Tensile Properties

Tensile properties of TPA and NC films were evaluated in an INTERMERIC iM50 (city of Mogi das Cruzes-SP, Brazil) universal mechanical testing machine with a 5 kN load cell, tensile speed of 5 mm/min, and an initial distance between jaws of 50 mm. The films were cut into strips with a width of 25.0 mm and a length of 75.0 mm. The evaluated properties were tensile strength (σ), modulus of elasticity (*E*), and elongation at maximum strength (ε). Three replicates were performed for each film type.^33^

#### 2.2.13 Soil Burial Degradation

Film samples measuring 2.5 × 2.5 cm were buried in natural soil at a depth of 15 cm, with approximately 80% humidity at room temperature. The visual aspect of the films was monitored at 0, 3, 5, 7, 10, and 16 days of burial in the soil.^15^ In this study, two types of soil were used: one with 30% humidity, coded as soil A, and one with 20% humidity, coded as soil B. After the end of each evaluation period, digital images were acquired to evaluate the visual aspect of each buried film.

#### 2.2.14 Application as Bread Packaging

Films measuring 10 × 6 cm were used to store pieces of bread (2 × 2 cm). The pieces of bread were packaged in TPA films with and without ZnO NPs, and then the packages were sealed. The presence of microorganisms such as fungi in the bread packaged in the films was visually monitored at 0 and 7 days.^34^ The bread used in this test was sourced from a local market in Ananindeua, Brazil.

#### 2.2.15 Statistical Analysis

The data collected from the films were analyzed to identify any statistically significant differences. The measurements were analyzed by analysis of variance (ANOVA) using Duncan’s test with a significance level of 5% (*p* < 0.05).

## 3 Results and Discussion

### 3.1 Fourier Transform Infrared (FTIR) Spectroscopy

TPA/ZnO NC films were obtained satisfactorily by a solvent casting method. In general, the films presented a homogeneous character, smooth surface, and absence of bubbles (Figure 2). For the TPA film, a transparent appearance was obtained, in contrast to the TPA/ZnO NCs films, which presented a translucent appearance. The low transparency for the TPA/ZnO NC films was attributed to the presence of ZnO NPs in the polymer matrix,^35^ as described in the transparency results (Section 3.8).

**Figure 2:**
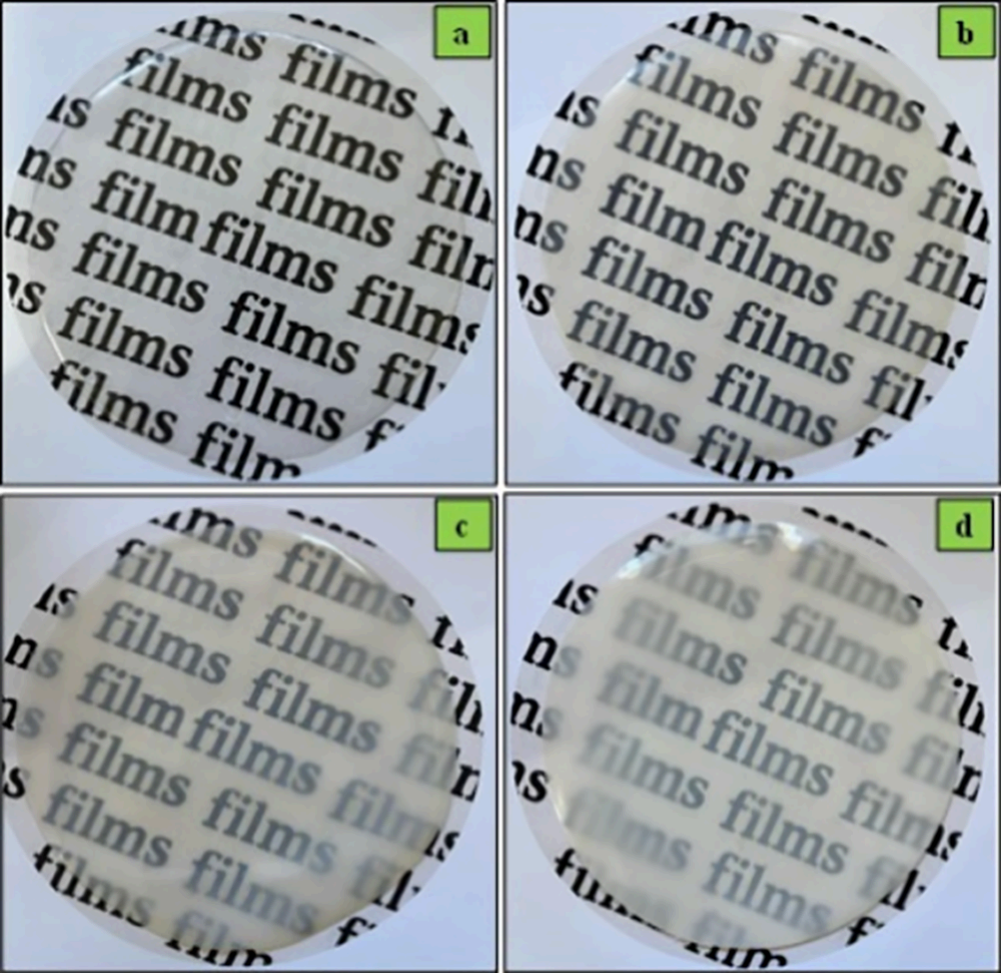
Visual appearances for (a) TPA, (b) TPA/ZnO-1, (c) TPA/ZnO-3, and (d) TPA/ZnO-5.

The effect of added ZnO NPs on the chemical structure of starch was assessed by using FTIR. FTIR spectra for the films with and without ZnO NPs are provided in Figure 3. For the TPA film, the vibrational mode observed around 3306 cm^–1^ was attributed to the stretching of the O–H bond of polymer and plasticizer (glycerol) and also residual moisture.^15,36,37^ Additionally, a vibrational band at 2930 and 2978 cm^–1^ was associated with the stretching of C–H bonds in the arrowroot starch,^38^ and the band at 1647 cm^–1^ was attributed to the hydroxy bending vibrations from residual moisture.^39,40^ Furthermore, the band centered at 1005 cm^–1^ was associated with the C–O–C glycoside bond from the polysaccharide structure present in starch.^15^ These findings were corroborated by Daza et al.^41^ TPA/ZnO NC films exhibit a spectral behavior similar to that of the TPA film without the presence of new absorption bands. These results are in good agreement with those found by Phothisarattana et al.. In their study, it was reported that the FTIR spectrum for the PBAT/TPS film was very similar to the spectra obtained for the PBAT/TPS films with ZnO NPs.^32^ This trend was also found for CMC-PVA films containing ZnO NPs.^42^ Charoensri et al. also observed a similarity between the spectra of thermoplastic starch and thermoplastic starch with ZnO NPs functionalized with polyaniline.^43^ Our findings indicate the absence of chemical reactions and the maintenance of the chemical structure of the polymer matrix after the insertion of ZnO NPs.

**Figure 3:**
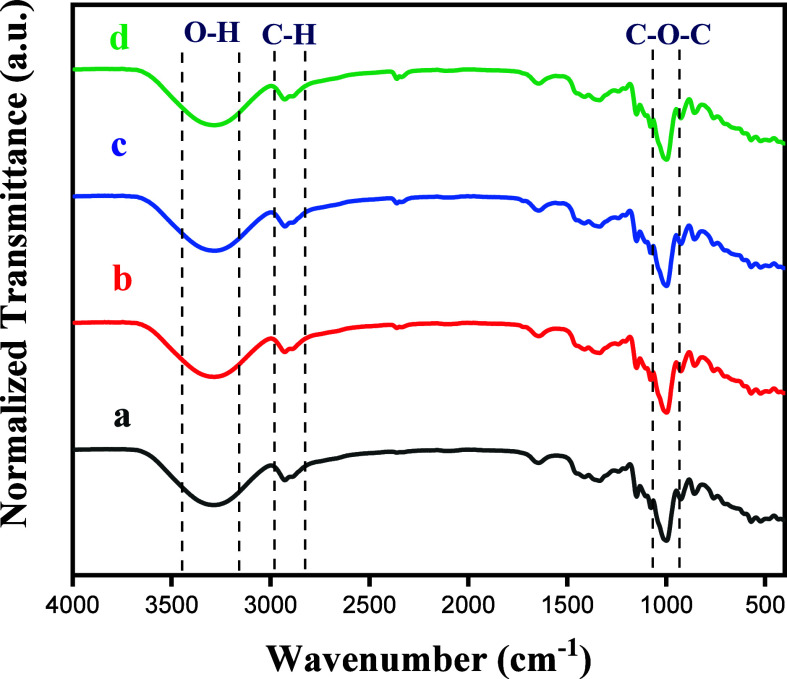
FTIR spectra for (a) TPA, (b) TPA/ZnO-1, (c) TPA/ZnO-3, and (d) TPA/ZnO-5.

### 3.2 Thermogravimetric Analysis (TGA)

The thermal stability of the TPA and NC films was investigated by acquiring TGA curves in an inert nitrogen atmosphere. TGA and derivative thermogravimetric (DTG) curves are provided in Figure 4. For all films, three mass loss events were observed. The first event occurred at a temperature lower than 100 °C and was attributed to the water adsorbed.^44^ In the second event in the range of 120 to 290 °C, the elimination of glycerol and water molecules took place.^15^ The third mass loss event occurred at around 300 °C; this last event was ascribed to the degradation and depolymerization of the carbon chains of arrowroot starch.^45^ These results are supported by studies of Rammak et al.,^39^ Guz et al.,^46^ and Ma et al.^47^

**Figure 4:**
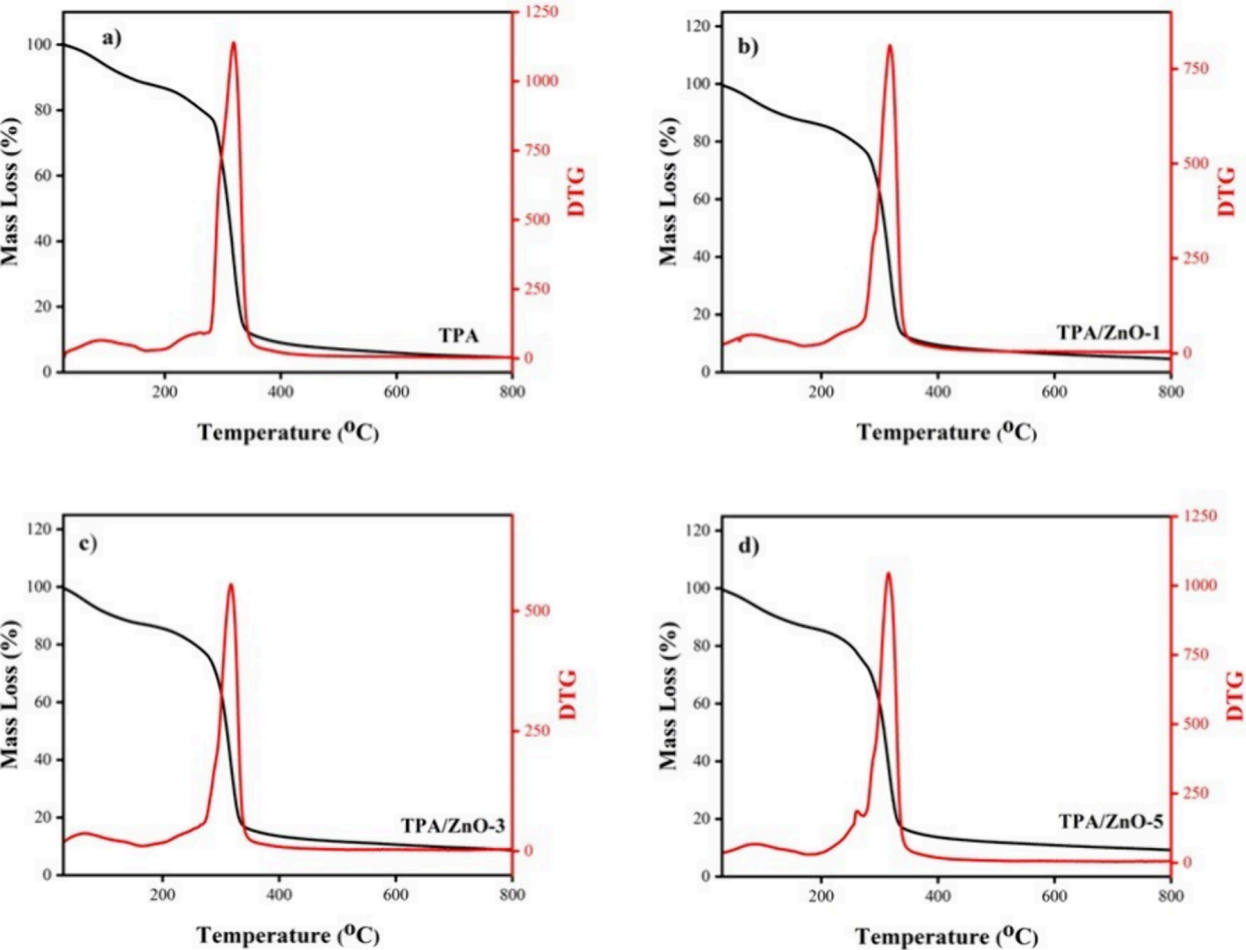
TGA (black line) and DTG (red line) curves for (a) TPA, (b) TPA/ZnO-1, (c) TPA/ZnO-3, and (d) TPA/ZnO-5.

NC films exhibited a thermal decomposition profile similar to that of the TPA film. The thermal properties of TPA and TPA/ZnO NC films were acquired by determining the temperatures *T*_10_ (10% mass loss), *T*_50_ (50% mass loss), and *T*_max_ (maximum temperature of degradation) and residual mass (%) at 800 °C (Table 3). Table 3 displays discrete variations for *T*_10_, *T*_50_, and *T*_max_ for all films; this means that the thermal stability of TPA film is preserved after the addition of ZnO NPs. Our findings are in good agreement with the previous study reported by Kodsangma et al. who evaluated the influence of the addition of ZnO NPs on the thermal properties of thermoplastic starch and starch/CMC films.^31^ Likewise, Paula et al. found the same thermal degradation profile, with slight changes, for PCL and PCL/ZnO NP films.^48^ Similar results for TGA were also reported for corn starch/sisal microfiber composite films.^38^ In contrast to our results, Mallakpour and Nouruzi reported a drastic reduction in the thermal stability of PCL after the addition of ZnO NPs treated with PVA. They stated that interactions between polymer chains are reduced due to the presence of these NPs.^29^ Our findings demonstrate that the addition of ZnO NPs to arrowroot starch did not cause a significant decrease in the thermal stability of the biopolymeric matrix.

**Table 3:** Thermal Properties for the TPA and TPA/ZnO NC Films

samples	*T*_5_ (°C)	*T*_10_ (°C)	*T*_50_ (°C)	*T*_max_ (°C)	residual mass (%) at 800 °C
TPA	86	138	310	318	4.49
TPA/ZnO-1	78	125	309	319	4.63
TPA/ZnO-3	68	115	311	316	8.60
TPA/ZnO-5	78	125	308	315	9.19

### 3.3 X-ray Diffraction (XRD)

The semicrystalline profile for TPA and TPA/ZnO films was determined by XRD. Figure 5 shows the diffractograms for the TPA and TPA/ZnO films. The TPA film did not exhibit the semicrystalline profile of arrowroot starch, which is indicative of a material without crystallinity. This finding aligns with the results reported by Vinhas et al. who observed that after starch gelatinization and film formation, the granular structure of the starch is destroyed, resulting in a predominantly amorphous material, as confirmed by our SEM results discussed in Section 3.5.^40^ However, characteristic peaks of arrowroot starch with low intensity were observed for TPA/ZnO-1 and TPA/ZnO-3. For TPA/ZnO NC films, characteristic peaks for ZnO are observed.^49^ As the NP content increases in the NC films, the intensity of the ZnO peaks increases. This increase was attributed to the greater amount of NPs, which favors the diffraction of these nanomaterials.^50^ Similar results were reported for NCs of chitosan/gelatin with Co-doped ZnO NPs.^51^ Our results indicate the formation of a thermoplastic material with characteristic peaks of ZnO NPs.

**Figure 5:**
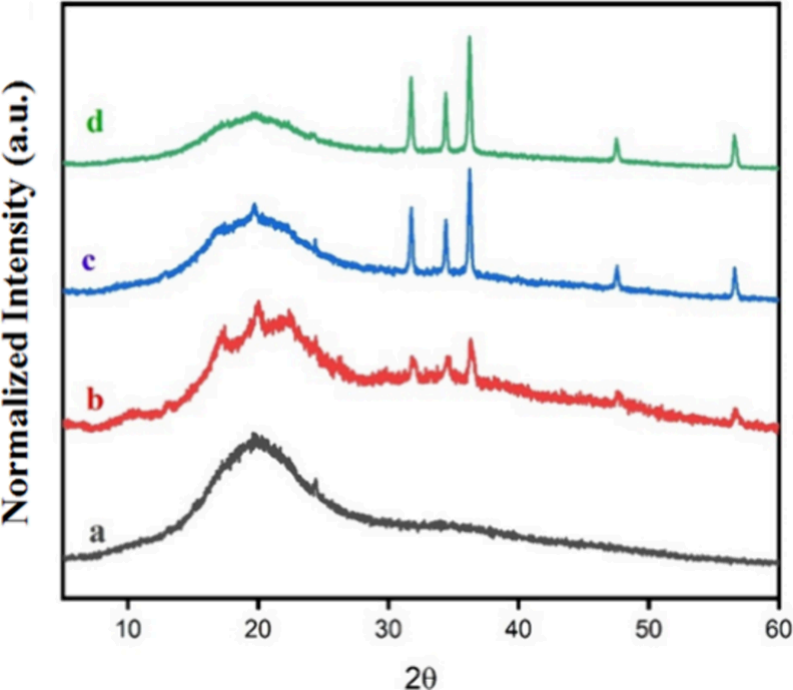
XRD for (a) TPA, (b) TPA/ZnO-1, (c) TPA/ZnO-3, and (d) TPA/ZnO-5.

### 3.4 Transmission Electron Microscopy (TEM)

Figure 6 reveals that the ZnO NPs used to obtain the NC films with arrowroot starch are in nanometric dimensions, which is a fundamental requirement to characterize a nanomaterial. TEM imaging results are consistent with previous studies reported in the literature.^52–54^

**Figure 6:**
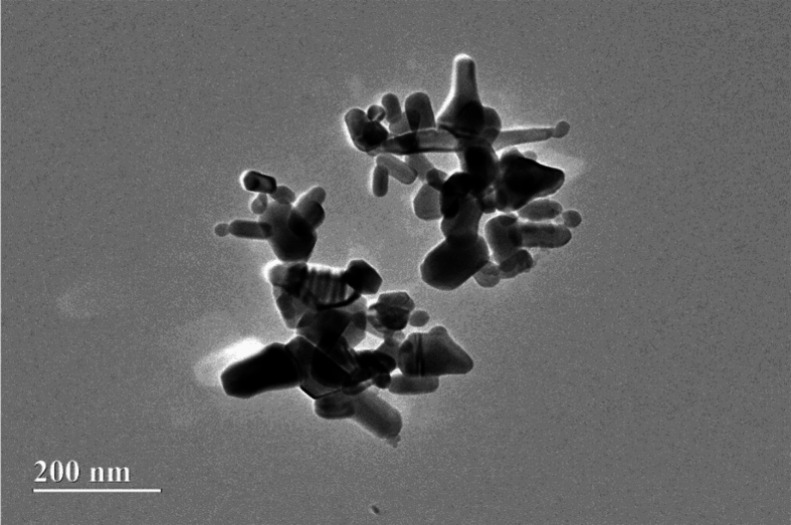
TEM image of ZnO NPs.

### 3.5 Scanning Electron Microscopy (SEM)

The morphology of arrowroot starch grains and ZnO NPs and the surface of the films were qualitatively evaluated by SEM Figure 7A illustrates the surface morphology for (a) arrowroot starch grains and (b) ZnO NPs, where starch grains have a granular morphology and ZnO NPs exhibit aggregates, characteristic of this material. From Figure 7B, it is possible to observe that the TPA film does not have a very smooth surface and cracks are present, which is attributed to the destruction of the arrowroot starch grains after plasticization with glycerol.^40^ In addition, Figure 7B demonstrates the presence of random aggregates for the TPA/ZnO NC films. These aggregates are a result of the incorporation of ZnO NPS into the arrowroot starch matrix.

**Figure 7:**
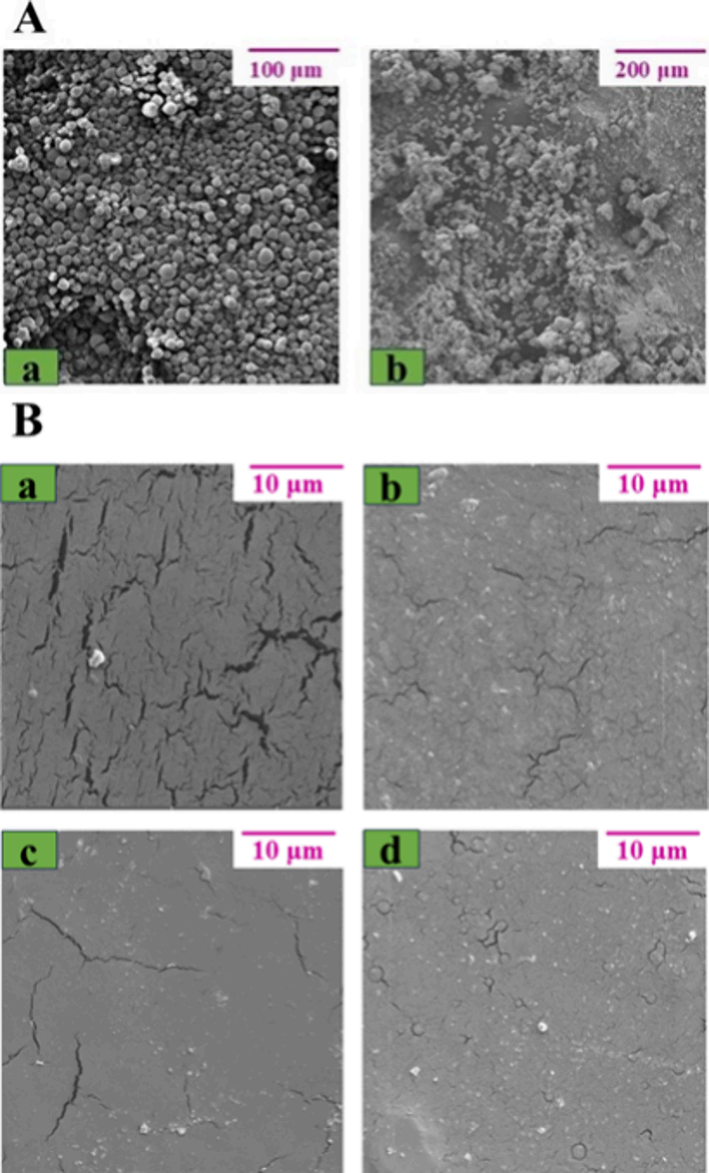
(A) SEM images for (a) arrowroot starch grains and (b) ZnO NPs. (B) SEM images for (a) TPA, (b) TPA/ZnO-1, (c) TPA/ZnO-3, and (d) TPA/ZnO-5.

### 3.6 Film Thickness

Thicknesses for TPA, TPA/ZnO-1, TPA/ZnO-3, and TPA/ZnO-5 are demonstrated in Figure 8a. The absence of significant statistical variations was observed for the average values of thickness between all samples. This behavior indicates that the addition of ZnO NPs did not affect the thickness of the NCs films. In general, thicker films have higher tensile stresses. This result is in agreement with previous studies.^15,47,48^ In support of this finding, the addition of montmorillonite (MMT) and ZnO NPs did not modify the thickness of CMC films.^55^

**Figure 8:**
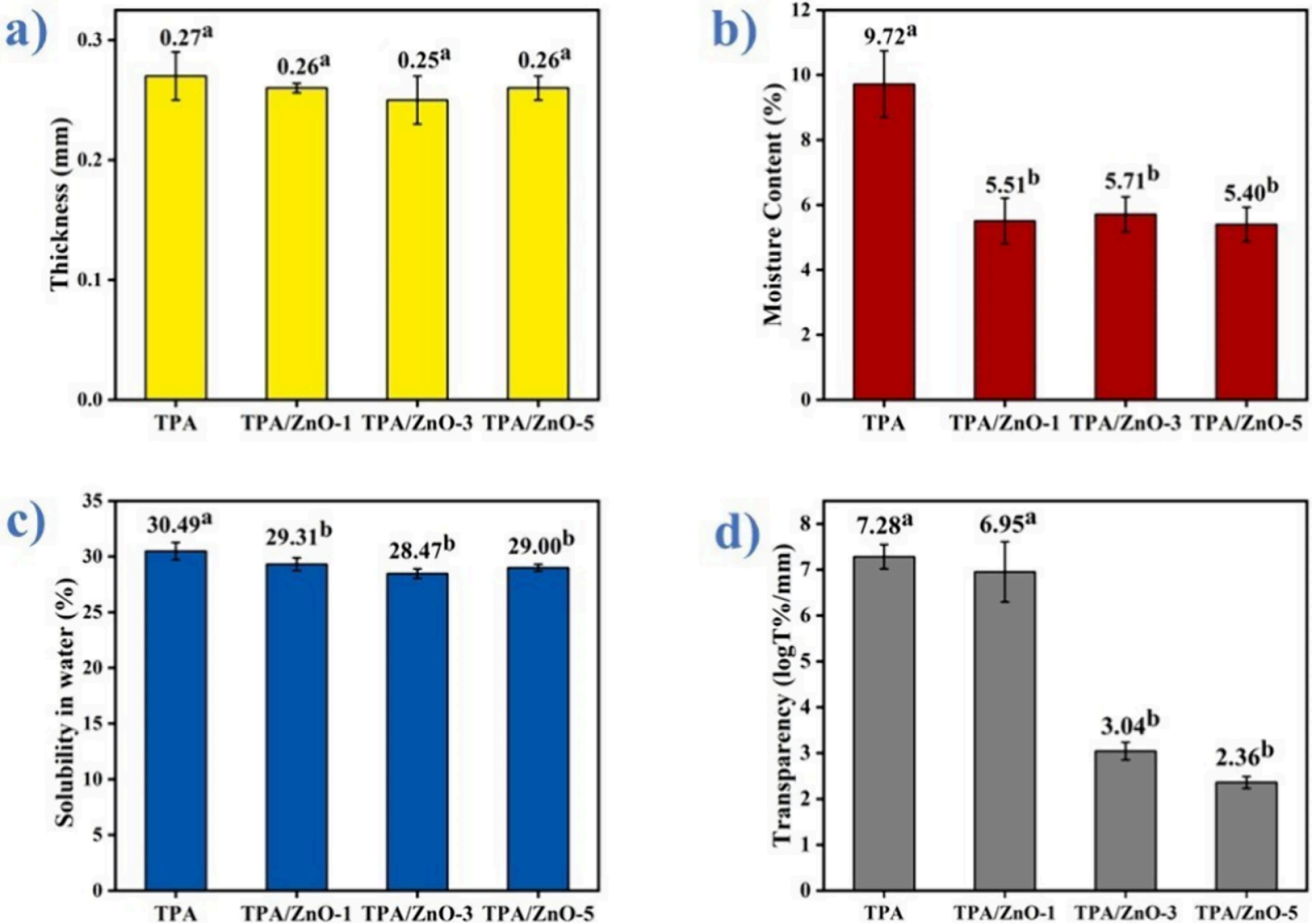
(a) Thickness, (b) moisture conte0t, (c) solubility in water, and (d) transparency for the films. Means followed by equal letters did not differ (*p* < 0.05) by Duncan’s test.

### 3.7 Moisture Content and Water Solubility

The moisture content is important to maintain the quality of products that are contained in biopolymer matrix NC films.^42^ In our study, we determined the moisture content in triplicate using the gravimetric method. The addition of 1, 3, and 5% ZnO NPs provided a decrease in the moisture content compared to the value found for the TPA film (Figure 8b). This decrease was attributed to the good interfacial interaction between the ZnO NPs and the starch matrix, which hinders the interaction of water molecules with the NC film.^42^ Our results were corroborated by Guz et al., who found a decrease in moisture content after the addition of ZnO NPs in a thermoplastic starch matrix.^46^ Similar results were also obtained by Charoensri et al.^43^

The solubility of films is a crucial factor in preserving the moisture of products stored in the packaging. Figure 8c presents the results of the solubility tests conducted for TPA, TPA/ZnO-1, TPA/ZnO-3, and TPA/ZnO-5 films, which were performed in triplicate. A slight decrease was determined for films with ZnO NPs. These results are attributed to the interactions established between the starch chains and the ZnO NPs, which hinder the solubilization of the films in water.^42^ This decrease is important to maintain the humidity of products stored in packaging and also film stability.^46^ Similar results were reported for ZnO NPs with polybutylene adipate terephthalate/TPS films.^56^ These results demonstrated that the addition of ZnO NPs provided an effective interaction between the nanomaterials and the starch matrix, thus improving the water susceptibility.

### 3.8 Transparency

Figure 8d depicts the transparency results. The TPA film exhibited the highest transparency; however, the addition of ZnO NPs to the starch matrix decreased the transparency of the films. This decrease was proportional to the increase in the content of NPs added. Similar results were reported for the opacity of poly­(lactic acid)/ZnO NC films.^57^ These transparency values are consistent with the visual aspect of the films, where films with white appearances are obtained after addition of the NPs. On the other hand, this less transparent aspect did not prevent the visualization of the content coated by the NC films, as shown in Figure 2.

### 3.9 Swelling in Water

The swelling behavior in water for films used in food packaging is essential to preserve product quality.^58^ The average swelling values for the four films investigated are displayed in Figure 9. Swelling was analyzed by the amount of water absorbed into the films at previously determined times. The TPA films in contact with water had a gradual increase with an increasing time investigated. The swelling for TPA at 5 h was 548.37%. After 24 h, the film showed an increase in swelling to 783.47%, reaching 963.12% in 14 days. This behavior was attributed to the hydrophilic characteristic of the TPA film, consisting of arrowroot starch and glycerol, which have hydroxyl groups that facilitate interaction with water.^59^

**Figure 9:**
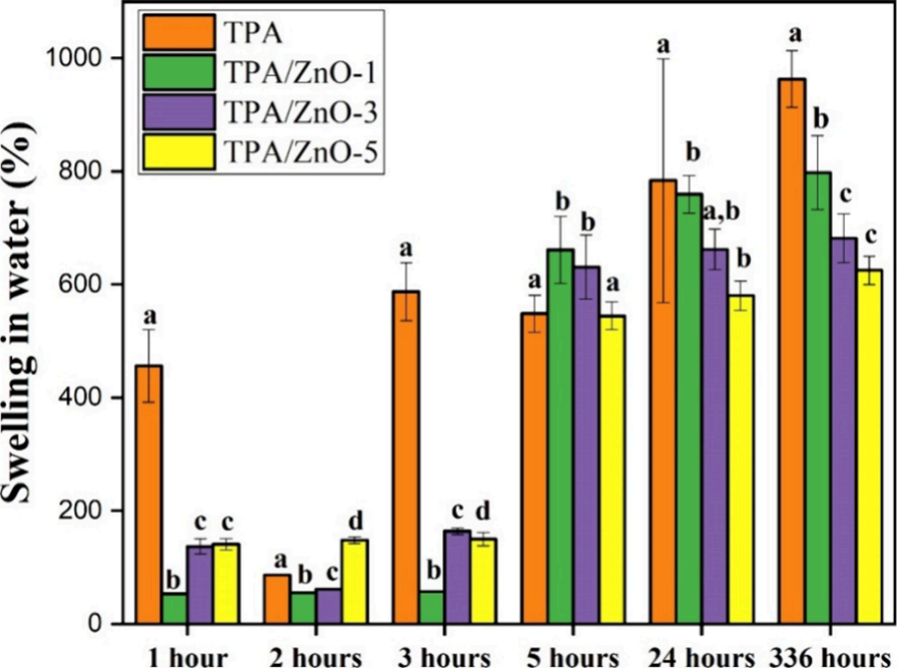
Swelling in water (%) for TPA, TPA/ZnO-1, TPA/ZnO-3, and TPA/ZnO-5. Means followed by equal letters did not differ (*p* < 0.05) by Duncan’s test.

Figure 9 also reveals a decrease in swelling values for TPA/ZnO-1, TPA/ZnO-3, and TPA/ZnO-5 in relation to the TPA film in each period evaluated. This decrease can be explained by the interactions established between the ZnO NPs and the arrowroot starch matrix, which does not facilitate the interaction of water with the NC film.^42^ However, our results demonstrate that after 14 days of contact with water, a relevant swelling is observed. In addition, the TPA/ZnO-5 film retained a lower water content, which is desirable for applications in products with high water content.^39^ These findings are consistent with moisture and solubility tests, where a decrease in humidity and solubility was attributed to the presence of ZnO NPs. Thus, our results demonstrate that the addition of ZnO NPs to the arrowroot starch matrix has a profound influence on the water retention capacity.

### 3.10 Tensile Stress Test

The influence of ZnO NPs on the tensile properties of the TPA starch matrix was evaluated by determining the tensile strength (σ), elongation at maximum strength (ε), and modulus of elasticity (*E*). Tensile property tests were performed in triplicate at a speed of 5 mm/min. Statistically significant variations were determined by Duncan’s test, with a significance level of 5%. Figure 10a–c displays the results for σ, ε, and *E* for the films, respectively.

**Figure 10:**
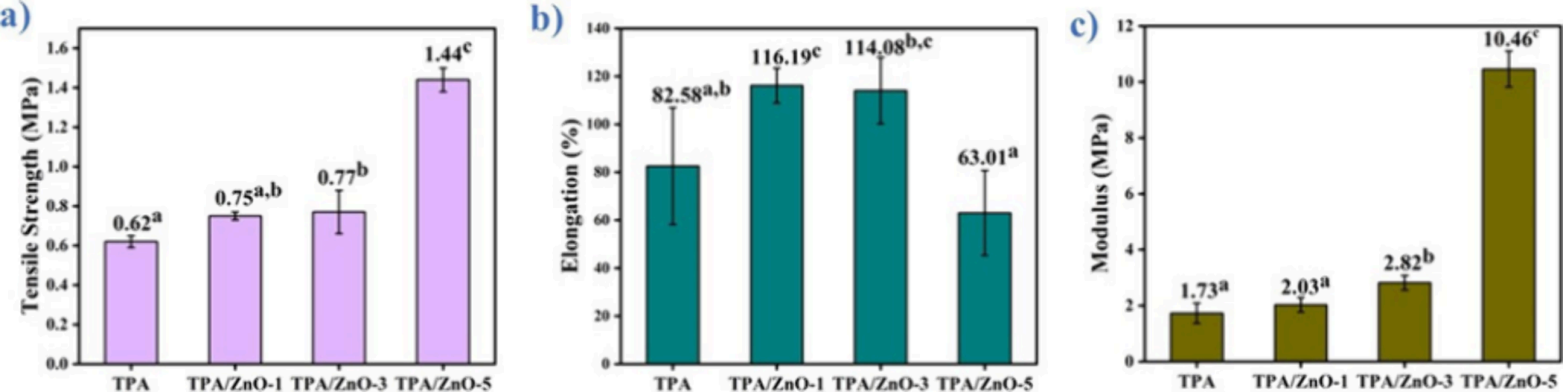
(a) Tensile strength (σ), (b) elongation (ε), and (c) modulus (*E*) for the films. Means followed by equal letters did not differ (*p* < 0.05) by Duncan’s test.

TPA film exhibited an average value of 0.329 MPa and 78.65% for σ and ε, respectively. The addition of ZnO NPs to the arrowroot starch matrix has increased the σ and ε for TPA/ZnO-1, TPA/ZnO-3, and TPA/ZnO-5 in relation to the TPA film. For TPA/ZnO-1, TPA/ZnO-3, and TPA/ZnO-5, the values for σ were equal to 0.360, 0.47, and 0.47 MPa, respectively. Additionally, the average values found for ε of TPA/ZnO-1, TPA/ZnO-3, and TPA/ZnO-5 films were equal to 90.72, 94.68, and 95.97%, respectively.

The addition of ZnO NPs promoted this increase due to their action as reinforcement of the starch matrix leading to more compatibility of these NPs with TPA. Furthermore, the values of σ and ε can be attributed to the random dispersion of nanoparticle aggregates within the polymer matrix, which enhances interfacial interactions between the components of the nanocomposite films, as illustrated in the SEM results (Section 3.5).^42^ The same trend observed for σ and ε was also observed for E; these findings occur due to the addition of NPs to the biopolymer. These results are in good agreement with Helmiyati et al., who investigated the influence of ZnO NPs on the tensile properties of CMC/PVA films.^42^ Additionally, Peighambardoust et al. investigated CMC-based active nanocomposite films containing montmorillonite (MMT) and Cloisite 30B clay NPs (modified with silver Ag and copper Cu ions). Their findings demonstrated that these films exhibited higher strain (ε) and modulus (*E*) values compared to pure CMC films.^60^ However, for glycerol plasticized pea starch/zinc oxide-starch bionanocomposites, a reduction for ε was found for higher NP contents. Thus, TPA/ZnO-5 films can be recommended for packaging applications because they have a good combination of the values of σ, ε, and *E*. In addition, previous studies have shown that NC films with 5% ZnO NP have good activity against both *Staphylococcus aureus* and *Escherichia coli* bacteria, which is suitable for increasing the shelf life of foods stored in these materials.^61^ Our findings demonstrate that the addition of ZnO NPs into the arrowroot starch matrix has a great influence on the values of σ, ε, and *E*.

### 3.11 Soil Burial Degradation

The biodegradability of the films was assessed through soil burial degradation. In this experiment, two types of soils, A and B, with moisture contents of 30 and 20%, respectively, were used. Figure 11a,b displays the results of degradation burial experiments for soil A and B. Figure 11a shows the presence of cracks for TPA, TPA/ZnO-1, TPA/ZnO-3, and TPA/ZnO-5 films buried in soil A at 3, 5, and 7 days. At 10 days of the experiment, no fragments of the films could be observed, thus making it impossible to continue the experiment. On the other hand, for the films buried in soil B, an incomplete degradation for all films was found at 16 days of the experiment (Figure 11b). Our results revealed complete degradation for the films buried in soil A at 10 days of the experiment. Biodegradation in soil occurs due to its humidity, the action of microorganisms present in the soil, or both conditions.^62^ In our experiments, the films buried in soil A were completely degraded within 10 days. This occurs because this soil has a higher moisture content. Higher moisture content facilitates the insertion of water molecules into the polymer chains and thus favors the degradation of the polymer matrix by soil microorganisms.^63^

**Figure 11:**
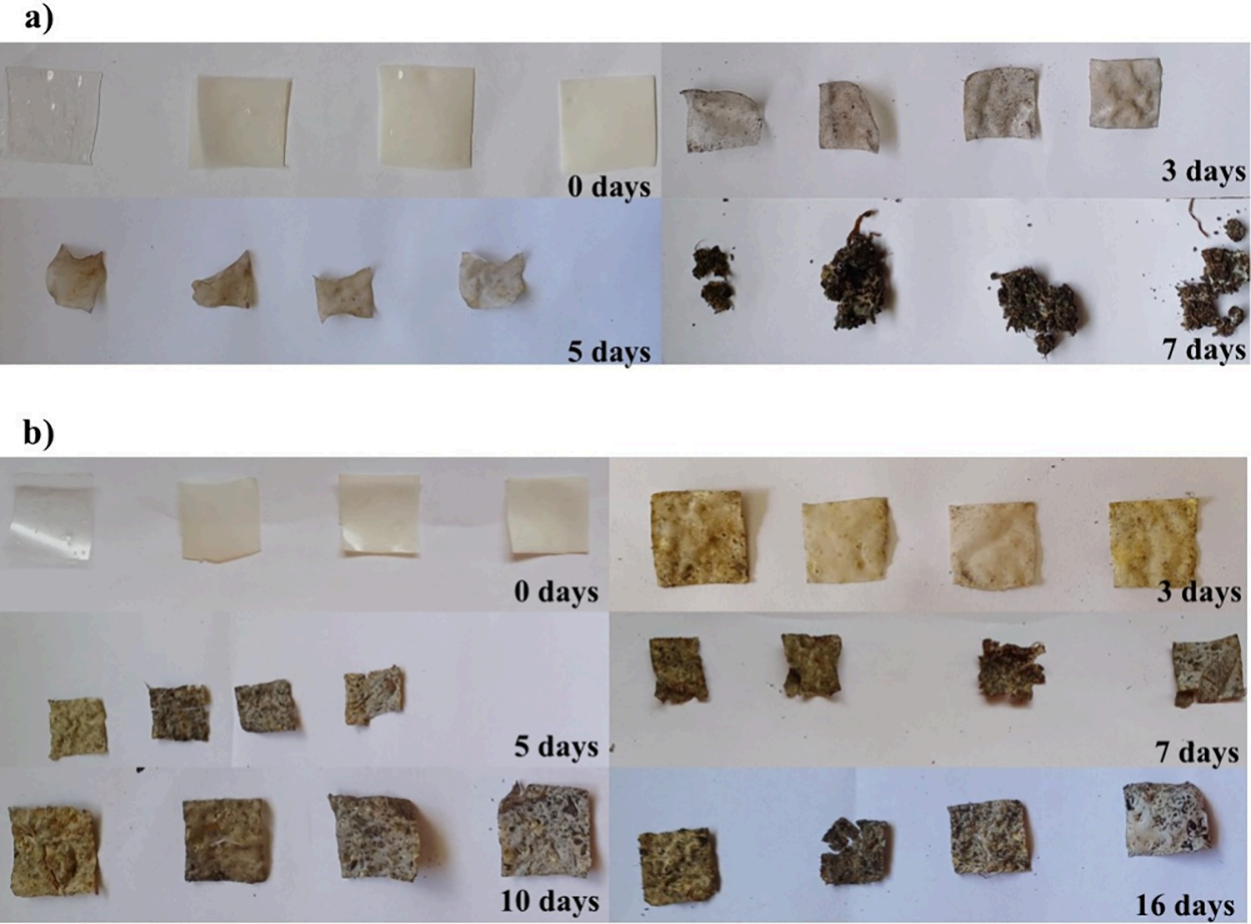
Soil burial degradation of TPA, TPA/ZnO-1, TPA/ZnO-3, and TPA/ZnO-5 (left to right) in (a) soil A and (b) soil B.

In contrast to these results, traces of the films buried in soil B were seen at 16 days of analysis. This behavior occurs because this type of soil has a lower moisture content that does not favor the action of soil microorganisms in the biodegradation process.^63^ Thus, our results demonstrate that soil degradation for all of the films is profoundly influenced by the soil moisture content. In addition, these findings reveal the biodegradability of TPA, TPA/ZnO-1, TPA/ZnO-3, and TPA/ZnO-5 films and their potential for use in the food packaging sector as a replacement for petroleum-based plastics.

### 3.12 Application as Bread Packaging

TPA, TPA/ZnO-1, TPA/ZnO-3, and TPA/ZnO-5 films were used to store bread pieces with dimensions of 2 × 2 cm. Figure 12 shows the appearance of the films stored in the films at 0 and 7 days of storage. Black and yellow spots, which are characteristic of fungal growth, were observed on bread stored in the film without ZnO NPs after 7 days. In contrast, no such spots were found on breads packaged in the TPA/ZnO-3 and TPA/ZnO-5 films during the same storage period. This behavior was attributed to the high antimicrobial activity of ZnO NPs added to the arrowroot films, which act to prevent the growth of microorganisms.^61^ This trend was observed for butter cakes packaged in cornstarch/poly­(butylene adipate-*co*-terephthalate) films with maltol and ethyl maltol.^14^

**Figure 12:**
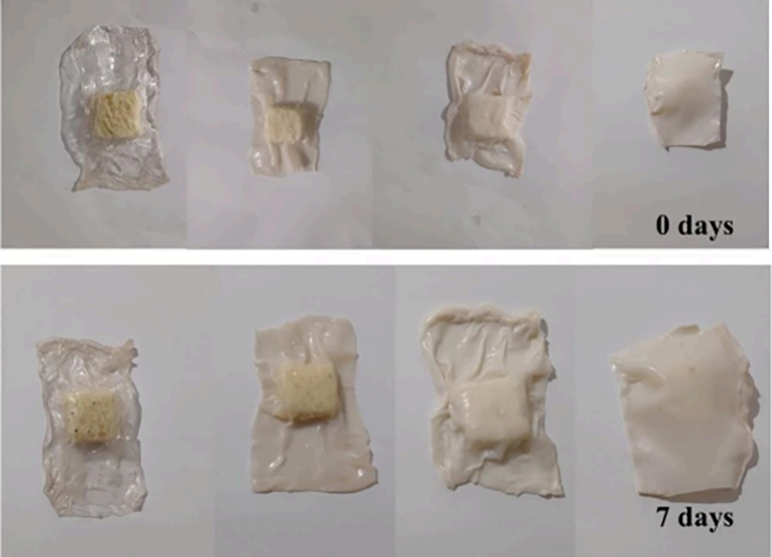
Bread pieces packed in TPA, TPA/ZnO-1, TPA/ZnO-3, and TPA/ZnO-5 films (left to right) at 0 and 7 days.

## 4 Conclusions

TPA/ZnO NC films containing 1, 3, and 5% ZnO NPs, plasticized with glycerol, were successfully produced using the solvent casting method. Overall, the films exhibited a homogeneous appearance, a smooth surface, and no bubble formation. However, as the concentration of nanoparticles increased, a noticeable decrease in the transparency was observed. FTIR spectra indicated that the polymer structure remained intact after incorporation of ZnO NPs. TGA curves showed that the thermal stability of arrowroot starch was not compromised by the presence of ZnO NPs. Characteristic peaks corresponding to ZnO NPs were detected in the diffractograms of the nanocomposites, with their intensity increasing proportionally to the amount of ZnO NPs included. SEM images revealed a random distribution of ZnO NPs within the starch matrix. The NC films exhibited improved water susceptibility, as highlighted by moisture and solubility tests. In the case of TPA/ZnO-5, there was a notable enhancement in tensile strength, increasing from 0.62 to 1.44 MPa, and in modulus, rising from 1.73 to 10.46 MPa compared to the TPA film. This enhancement in the mechanical properties was due to the formation of interfacial interactions between the components of the NC films. Our findings indicate that the degradation of all films through soil burial is influenced by the soil moisture content. Additionally, no black or yellow spots characteristic of fungal growth appeared on bread stored in TPA/ZnO-3 and TPA/ZnO-5 films after 7 days. Our results indicate that TPA/ZnO-5 demonstrated a favorable combination of σ, ε, and *E* and also exhibited soil burial degradation.
